# Clinical Experiments, &c.

**Published:** 1781-02

**Authors:** 


### II. *Clinical Experiments*, &c. *By* Francis Home, M. D. [*Continued from Page* 20.]

[*Continued from Page* 20.]

IN his twelfth fection our author prefents us with his experiments on fome antiparalytic remedies. In fix cafes of palfy he tried the Arnica montana, or leopard's bane, but without much fuccefs, fo that he leaves it to future trials, before he can fubfcribe to the accounts that have been publifhed of this remedy at Vienna: he can only fay, that from the ftimulus it produces in the primæ viæ, and on the affected mufcles, it feems to give hopes that it may perhaps be of fome fervice. The patients ufually took from one to three drachms of the leaves infufed in a pint of boiling water in the courfe of the day. In fome it occafioned naufea and purging, but in two of the patients it produced no fenfible effects. We may obferve here, that the flowers, and not the leaves, of the Arnica, have been the moft ftrongly recommended in paralytic cafes; and it may be farther objected by thofe who read Dr. Home's experiments, that the medicine was not given in a fufficient quantity, or continued long enough, to allow it a fair trial.

In a palfy of the lower extremities he whipped the affected part with nettles: they produced a burna burning heat and fevere pains, but no relief. In three other cafes he tried the hot bath, but with no better fuccefs; and hence he infers, that as natural hot baths have been found to be powerful remedies in this difeafe, they probably operate from mephytic air and other impregnations which common water has not. We cannot fubfcribe to this opinion, as we have always thought that the ftimulus of hot mineral baths, which renders them ufeful in palfy, is owing chiefly, if not folely, to their heat.

In the thirteenth fection we find an account of the effects of ol. terebinthinæ and honey in the fciatica, a remedy originally recommended by Dr. Cheyne in his medico-philofophical works. Two drachms of the oil are to be mixed with an ounce of honey, and the patient is to take a tea-fpoonful of this linctus morning and evening, fwallowing a draught of warm water after each dofe. Seven cafes are related by Dr. Home, five of which it cured, the other two were relieved by it. He informs us likewife that he has long ufed it with great fuccefs in private practice. Of the feven patients, whofe cafes are here mentioned, five were men, and only two women; and from this circumftance, the author (we fear too haftily) concludes that men are more more ſubject to this diſeaſe than women; nor does he ſeem to have any better ground for aſſerting, in the next page (when pointing out the difference between the ſciatica and rheumatiſm) that the rheumatiſm attacks women oftener than men in the proportion of 3 1-half to 1, merely becauſe of 18 rheumatic patients whom he ſaw in the clinical ward, 14 happened to be females. Dr. Home conſiders the ſciatic nerve as the ſeat of the ſciatica. The ſenſible operation of the remedy, we are told, is various. It often produces a heat in the ſtomach, and diminiſhes the appetite. It heats the part, and raiſes a peculiar ſenſation of pain there. In two of the patients it proved diuretic, and in a third it brought on a ſtrangury, by being given in too great a quantity. In one of the caſes it performed a cure in four days, but in ſome it required fourteen. He finds it of uſe only in the pure ſciatica.

In the fourteenth ſection Dr. Home relates the effects of a liniment in the lumbago. This application, which he formerly recommended in his *Medical Facts*, is directed to be prepared in the following manner:

℞. Camphor. ℈j

Diſſol. in ol. terebinth. ℥ij

Sal.

Sal. c. c. gr. xv.

P. ſem. Cymin. ʒij

Dein adde ung. nervin. ℥ſs

Sapon. nigr. com. ℥j

M. f. linim. extend. ſuper alutam et applic. lumbis.

Four caſes are deſcribed in which this remedy proved of uſe. In one of theſe, bliſters, and even the actual cautery, had been tried to no purpoſe; the above liniment relieved, though it did not cure this patient. After all, however, we cannot conceive why it can be preferable to any other ſtimulating application. A plaſter of burgundy pitch would perhaps prove equally efficacious.

In the fifteenth ſection our author deſcribes a caſe of iſchuria renalis, that terminated in death. Neither Morgagni nor Valſalva ever met with an inſtance where the ſuppreſſion of urine was owing to a diſeaſe of both the kidneys, without the bladder being affected. Dr. Home obſerves, that there was no ſwelling in the lower part of the abdomen; that the patient had no deſire to paſs urine; and that the inſpection of the body ſhewed that none had been ſecreted. After this hiſtory follows one of iſchuria veſicalis, attended with this uncommon circumſtance, that the patient died from a diſtenſion of the bladder, although he continued through the whole courſe of the diſeaſe to paſs his urine, even in more than a natural quantity. The diſeaſe upon diſſection was found to have been occaſioned by a preternatural thickening and enlargement of the bladder. Dr. Home relates another caſe of Iſchuria Veſicalis, which ſeemed to be owing to a ſchirrous affection of the bladder. The patient remained in the clinical ward, after the Doctor's attendance there was finiſhed, ſo that we are not informed of the fate of this patient. After this, our author mentions a caſe of Iſchuria Urethralis that happened in conſequence of a gonorrhœa.

The ſixteenth ſection is allotted to diabetes, of which our author gives the following definition: "Urina aucta et ſubdulcis; ſitis perpetua; "cutis arida et plerumque ſquamoſa." Two caſes are related, the ſymptoms of which were ſimilar. One of the patients paſſed from 12 to 15 pints of urine in four-and-twenty hours, and his urine exceeded his drink uſually about two pints; the other voided ſomewhat leſs in quantity, but more in proportion to his drink; when he drank 4, he paſſed 12 pints a day; when he drank 3, he paſſed 10 pints, ſo that his urine always exceeded his drink by 8, or at leaſt 7 pints. In each patient more urine was conſtantly voided from 12 at noon to 12 at night, than during the other 12 hours. The urine of both had a ſweet taſte, but afforded no oil on its ſurface, nor was it coagulable by fire or ol. vitrioli. The urine of one of the patients, evaporated to an extract by Dr. Black, afforded 1 1-half oz. from each pound of a brown ſaccharine matter, which had a weak ſaltiſh taſte. That of the other patient, treated in the ſame way, yielded but one ounce for each pound, of a ſubſtance reſembling coarſe brown ſugar, with a ſaccharine and urinous ſmell, and a weak and ſalt taſte. Fermented by means of yeaſt, the urine of both produced what our author calls, "a tolerable "ſmall beer." This circumſtance, he obſerves, proves the contents of the urine to be moſtly of a vegetable nature, as no animal fluids are capable of the vinous fermentation. In both patients the appetite was much greater than in good health; their ſkins were dry, and that of one of them ſcaly. Neither of the two ever ſweated. One of theſe patients, after having been ten weeks in the clinical ward, was diſmiſſed in the ſame ſtate he came in; the other died there. Diſſection tion did not seem to throw any new light on the nature of the disease. One of the kidneys was larger and softer than usual. No lacteals were observable about the neck of the bladder.

In the seventeenth section we meet with experiments upon some remedies used in dropsy. The first in the list is cremor tartari, a medicine recommended several years since by Menghini in the *Comment. Bonon.* Tom. 4. as a cure for dropsy when given from ℥iv to ℥vj a day. In sixteen instances of its good effects related in that work, twenty and sometimes forty days elapsed before it had any visible effect in increasing either stool or urine; but afterwards the belly became loose, and the urine thick, bilious, and sabulous. Dr. Home describes twenty cases in which this medicine was administered; of this number, we are told, thirteen were cured. After reviewing the cases, our author concludes that this remedy is most to be depended on in anasarca, next to that in ascites, and least of all in hydrothorax, though it seems that of 4 patients in the latter disease, two were cured by it. The general effects of this medicine were now and then, though rarely, to have excited vomiting: it usually purged twice or thrice a day with ease, but in some of the patients it seemed to have a contrary effect. It commonly increased the quantity of urine, though not near so much as the bac. junip. squills, &c. and in some, the discharge of urine was not sensibly or but little augmented. The urine, except in two cases, was not thick and sabulous, as in Menghini's patients, but paler and clearer than natural. During the use of it, the appetite mended, and the thirst, heat, and fever diminished. They were a little thinner at the end, but not so much so as Menghini describes. It was given from ℥ß. to ℥ij. a day, dissolved in about twenty times its quantity of water. The second remedy of which Dr. Home made a trial, was the *scilla exiccata.* He prescribed it in ten cases, in seven of which it succeeded, but he found it useful only when it excited vomiting. Three or four grains three times a day were generally found sufficient to produce this effect. In three other cases our author tried the effect of issues in anasarca, and found them useful.

In his eighteenth section, our author describes his experiments upon some remedies used in the amenorrhoea. In six cases he tried the compression of the crural artery, recommended by Dr. Hamilton in the Phys. and Lit. Essays, in order to produce a temporary plethora in the uterus. But this method was succeeded by an appearance of the menſes only in one of the ſix patients. In moſt of the patients the tourniquet was applied to both thighs for an hour, and in one caſe was continued till the thighs were diſcoloured. It generally quickened the pulſe, and produced head-ach, vertigo, dyſpnœa, and pains in the ſtomach and abdomen. In three other patients V. S. was tried and ſeemed to be uſeful. Dr. Home gave pulv. ſabin. in five caſes, and with good ſucceſs in three of the five. This remedy, which has long been conſidered as a powerful emmenagogue, he has found a uſeful and ſafe medicine. Half a drachm is the doſe in which he has commonly given it. Our author was induced to give the rubia tinctorum in amenorrhoea upon the authority of Tournefort, who ſays, that "it ſtrongly provokes the courſes." Nineteen caſes are related, in which from ℈ſs to ʒj. of it in powder were given four times a day. Fourteen of theſe patients we are told were cured, although the medicine produced hardly any ſenſible effect, never having quickened the pulſe or increaſed the inflammatory ſymptoms. Our author's encomiums on this medicine induced us to give it a trial, but we experienced no good effect from it, and in one patient it conſtantly produced vomiting.

The nineteenth ſection relates to the herpes, or Lepra Græcorum, a diſeaſe that frequently occurs, eſpecially amongſt the lower ſort of people. In theſe caſes the ſkin is attacked with cruſty or ſcaly ſcabs, ſometimes dry, ſometimes ulcered, and generally diſpoſed in cluſters. With ſome patients labouring under complaints of this kind, Dr. Home tried the tincture of cantharides, combined with muc. gum. arabic. but without having been able to perform a complete cure with it, although the ſymptons were alleviated by it. He was not more fortunate with the inner bark of the elm given in the manner recommended by Dr. Lyſons. Our author tried it in four caſes; one of the patients was diſmiſſed, cured, after taking it, but as it produced no viſible effects, he doubts its efficacy. The author tried viper broth, and an electuary prepared from the boiled fleſh of vipers, in three other caſes, but his experiments with this remedy are far from being ſatisfactory. To four of his patients he gave a decoction of ſarſaparilla, but without any apparent good effect. He was more fortunate with Plummer's pill, having cured with it ſeven out of nine patients. He obſerved that it rarely produced nauſea; that it looſened the belly, in nearly half of the patients, and that it was attended with a moiſture each night in a great number; a gentle ſpitting was excited in two, and a ſalivation in a third. Our author alſo tried the acidum vitriolicum, which has of late been ſo much extolled in Germany as a remedy for cutaneous affections: he gave it to two patients but without ſucceſs. We ourſelves have preſcribed this medicine in the largeſt doſes, and for a long continuance, to ſeveral patients, but never experienced any good effects from it.

In his twentieth ſection our author relates his experiments on the vermifuge effects of the *Spigelia Marylandica* or Indian pink, which is the root of a plant that grows in the low grounds of South Carolina: it was diſcovered about the year 1740 to the Europeans by the Indians, as a vermifuge medicine. From eight trials with this remedy, Dr. Home concludes that it is an effectual and valuable medicine, as it always carried off the ſymptoms occaſioned by worms. He obſerves that it takes ſix, ſometimes eighteen days to remove thoſe ſymptons; that children of eight years may take 10 grains twice a day, and adults ʒſs four times a day, with ſafety. In ſpeaking of the ſymptoms of worms, our author remarks that a ſwelling of the noſtril and upper lip is a more conſtant one than any of the reſt; that it accompanies the diſeaſe when there is no ſuſpicion of

ſcrophula,

ſcrophula, and retires with the worm ſymptoms when they ariſe in ſuch a habit; and that, where this ſymptom, which is often not to be obſerved, appears in the ſcrophula, it probably ariſes from worms. This obſervation ſeems to us to ſtand greatly in need of confirmation by the experiments of others.

The twenty-firſt ſection relates to Mezereon, the root of which he found efficacious in ſeven trials. We are told that it reduced ſchirrous ſwellings which had appeared incurable, and that it cured many of them after a courſe of mercury had failed. Two drachms to a bottle, which was drank in the courſe of a day, proved a ſufficient doſe; when another ʒ ſs was added, it produced too great ſickneſs.

In the twenty-ſecond ſection our author deſcribes the effects of the *Verbaſcum* in Diarrhœa. The white or cow's lungwort was the ſpecies he preferred; its taſte is ſomewhat rough, though it is not aſtringent: it has been much uſed, it ſeems, in Italy, as a pectoral. In four caſes of diarrhœa it appeared to poſſeſs conſiderable efficacy; it was given in decoction; ℥j of the leaves were generally boiled in ℔ ij of water, and the patient drank ℥iv every third hour. Our author however thinks that proportion too little, and would rather recommend two ounces. As it diminiſhes minishes or stops diarrhœas of an old standing, and often eases the pains of the intestines; he thinks it acts by its emollient qualities, obviating irritability, and perhaps by its being gently astringent. Linnæus says, that when mixed into a paste with flour and thrown into water, it stupefies fishes so that they may be taken with the hand; it would seem therefore to possess an anodyne property.

In the twenty-third section we meet with experiments upon the antihemorrhagic effects of the *cucurbitulæ sine ferro*, or dry cupping, an application much used by the ancients, and particularly recommended by Hippocrates in the menorrhagia, who orders the application to be made to the breasts: this was accordingly done by our author in three cases, and with success; the glasses were ordered to be applied generally twice a day, and to be continued for a quarter of an hour. He observes that they have a sudden and powerful effect in stopping hæmorrhages from the uterus: he likewise found them of use in a case of hæmatemesis.

In the last section our author treats of two Lithontriptics, caustic lie and mephitic air. He tried the first of these in four cases, but without success, though from experiments made with the urine of some of the patients he found it impregnated

 with with the qualities of the caustic alkali, and possessing some power of dissolving stones. He is therefore unwilling to discard this remedy too hastily. He tried the mephitic air in one case, but without any good effect. The patient drank two pints of mephitic water a day, and had 4 ounces of it injected into his bladder every morning and evening. He continued in this course for 26 days without any alleviation of his symptoms, and was afterwards cut, and had a large stone taken from him.

